# Expression, correlation, and prognostic significance of different nicotinic acetylcholine receptors, programed death ligand 1, and dopamine receptor D2 in lung adenocarcinoma

**DOI:** 10.3389/fonc.2022.959500

**Published:** 2022-08-22

**Authors:** Krishnendu Pal, Tabish Hussain, Hao Xie, Shenduo Li, Ping Yang, Aaron Mansfield, Yanyan Lou, Shantanu Chowdhury, Debabrata Mukhopadhyay

**Affiliations:** ^1^ Department of Biochemistry and Molecular Biology, Mayo Clinic, Jacksonville, FL, United States; ^2^ Genomics and Molecular Medicine Unit, CSIR-Institute of Genomics and Integrative Biology, Mathura Road, New Delhi, India; ^3^ Division of Medical Oncology, Mayo Clinic, Rochester, MN, United States; ^4^ Division of Hematology and Medical Oncology, Mayo Clinic, Jacksonville, FL, United States; ^5^ Department of Quantitative Health Sciences, Mayo Clinic Scottsdale, AZ, United States; ^6^ Integrative and Functional Biology Unit, CSIR- Institute of Genomics and Integrative Biology, New Delhi, India; ^7^ Academy of Scientific and Innovative Research, CSIR- Institute of Genomics and Integrative Biology, New Delhi, India

**Keywords:** lung cancer, NSCLC, smoking, nAChR, DRD2, PD-L1

## Abstract

**Objective:**

The objective of this study is to evaluate the expression of different nicotinic acetylcholine receptors (nAChRs), programmed death ligand-1 (PD-L1), and dopamine receptor D2 (DRD2) as prognostic factors in lung cancer and any correlation among them. Since all of the above genes are typically upregulated in response to smoking, we hypothesized that a correlation might exist between DRD2, PD-L1, and nAChR expression in NSCLC patients with a smoking history and a prediction model may be developed to assess the clinical outcome.

**Methods:**

We retrospectively analyzed samples from 46 patients with primary lung adenocarcinoma who underwent surgical resection at Mayo Clinic Rochester from June 2000 to October 2008. The expression of PD-L1, DRD2, CHRNA5, CHRNA7, and CHRNA9 were analyzed by quantitative PCR and correlated amongst themselves and with age, stage and grade, smoking status, overall survival (OS), and relapse-free survival (RFS).

**Results:**

Only PD-L1 showed a statistically significant increase in expression in patients older than 65. All the above genes showed higher expression in stage IIIB than IIIA, but none reached statistical significance. Interestingly, we did not observe significant differences among never, former, and current smokers, but patients with pack years greater than 30 showed significantly higher expression of CHRNA9. We observed a strong positive correlation between PD-L1/DRD2, PD-L1/CHRNA5, and CHRNA5/CHRNA7 and a weak positive correlation between DRD2/CHRNA5 and DRD2/CHRNA7. Older age was independently associated with poor OS, whereas lower CHRNA7 expression was independently associated with better OS.

**Conclusions:**

We observed strong positive correlations among PD-L1, DRD2, and some of the nAChRs. We investigated their prognostic significance in lung cancer patients and found CHRNA7 to be an independent prognostic factor. Overall, the results obtained from this preliminary study warrant a large cohort-based analysis that may ultimately lead to potential patient-specific stratification biomarkers predicting cancer-treatment outcomes.

## Introduction

Lung cancer is the leading cause of cancer-associated mortalities irrespective of gender in the United States and worldwide (1, 2). Approximately 85% of lung cancer falls under non-small-cell lung cancer (NSCLC), while the rest is categorized as small-cell lung cancer (SCLC) (3). Tobacco smoking is the primary risk factor for lung cancer; SCLC is exclusively seen among smokers, whereas approximately 80-90% of NSCLC is smoking-related (4, 5). Traditionally, nicotine has been considered addictive only and not carcinogenic. However, studies have shown that nicotine promotes proliferation, migration, invasion, and survival *in vitro* and tumor growth and metastasis *in vivo* in cancers of different origins, including lung cancer (6–15). The primary underlying mechanism behind the tumor-promoting activity of nicotine is exerted through the upregulation and activation of nicotinic acetylcholine receptors (nAChR) (10, 16–18). Acetylcholine (Ach) is the endogenous ligand of nAChRs, but nicotine can displace Ach due to its higher affinity towards these receptors, thereby activating downstream tumor-promoting signaling cascades (19–21).

The nAChRs consist of five subunits spanning the plasma membrane and are organized symmetrically around a central ion pore (22, 23). They act as ligand-gated ion channels facilitating calcium flux and release of neurotransmitters in neuronal cells and neuromuscular junctions or growth factors such as VEGF in epithelial and endothelial cells (23–25). To date, 17 nAChR subunits (α1-α10, β1-β4, δ, ϵ, and γ) have been identified in vertebrates, although only a few of them (α3-α7, α9, β2, and β4) have been studied in the context of cancer (26).

Dopamine receptors (DRD1–5) are typically observed in the brain (27, 28), but have also been shown to express in the tumor microenvironment, where they play vital roles in tumorigenesis and cancer progression (29–34). We initially unveiled that DRD2 is essential for dopamine-mediated selective inhibition of VPF/VEGF-induced vascular permeability and angiogenesis (35). DRD2 is implicated in lung cancer (36–39), and DRD2 agonists demonstrate significant growth inhibition in both NSCLC and SCLC (29, 31, 40, 41). Interestingly, some DRD2 variant genotypes have been associated with a higher affinity to smoking and a lower likelihood of smoking cessation, which has been proposed to be a probable cause behind the familial aggregation of smoking-related cancers (42, 43). DRD2 genetic polymorphisms have been associated with reduced bioavailability of dopamine and a higher risk of NSCLC among smokers (36). We also showed that DRD2 expression in tumors of lung cancer patients demonstrates a positive correlation with the extent of cigarette smoke exposure and the histological grade of the tumor (29). Several studies suggested potential cross-talk between DRD2 with nAChRs within the brain (44–49), but no conclusive evidence of their interaction in cancer has been observed till now.

The interaction of programmed cell death protein-1 (PD-1) with its ligands, namely programmed cell death ligand-1 and -2 (PD-L1, PD-L2), act as immune checkpoints by reducing functionality of effector T-cells in peripheral tissues, and preventing them from attacking the host cells during inflammatory response (50, 51). Cancer cells hijack this mechanism to evade immune surveillance and induce immune suppression by expressing PD-L1 or PD-L2 on their surface (52, 53). Immune checkpoint inhibition *via* antibodies against PD-1 or PD-L1 demonstrated improved therapeutic response in several types of cancer, including NSCLC (54–58). Although patient stratification based on PD-L1 expression improved the response rate compared to non-stratified patients (59–61), a response is not always dictated by their expression (62–66). Interestingly, recent studies demonstrated a strong correlation between PD-L1 expression and smoking status in NSCLC patients, where smokers with higher pack-years demonstrated a higher intensity of PD-L1 expression (67–69). However, the correlation between PD-L1 and nAChRs have not been well studied in NSCLC patients.

The primary goal of the present study is to determine whether the expression pattern of PD-L1, DRD2, and the genes encoding different nAChR subunits in NSCLC are affected by the age, stage, grade, and smoking status and whether they could explain the variability of the influence of tobacco smoking in response to therapy and survival in NSCLC. Towards this end, we have used real-time quantitative polymerase chain reaction (qPCR) to examine normalized mRNA expression levels of the above genes in surgical tumor samples from 46 NSCLC patients and examined their correlation with the said parameters as well as with every other gene in our study.

## Materials and methods

### Sample collection

We collected flash-frozen samples of lung tumor tissues surgically removed from 46 patients with primary lung adenocarcinoma. The patients were admitted to and underwent surgery at Mayo Clinic Rochester from June 2000 to October 2008. Detailed demographic analyses of the patients are provided in **Table 1**. To avoid the potential confounding impact of the treatment, none of the selected patients received neoadjuvant treatment before the surgery, although some of the patients were treated with chemotherapy or radiotherapy or both post-surgeries. Tumor grading was abstracted from chest pathologists’ diagnosis documented in Mayo Clinic medical records and categorized as well-differentiated, moderately differentiated, poorly differentiated and undifferentiated. Tumor staging was based on the TNM staging system 7^th^ edition (70). The Mayo Clinic Institutional Review Board reviewed the study protocol, and all patients signed written informed consent forms.

**Table 1:** Patient characteristics.

Characteristics	Patients (n=46)
Age at diagnosis (years)	65 (41–84)
PD-L1	11.3 (3-21.2)
DRD2	15.5 (8.9-25.9)
CHRNA5	10.2 (2.6-20.8)
CHRNA7	15.9 (6.7-26.8)
CHRNA9	17.8 (2.8-26.7)
Gender	
Female	22 (47.8%)
Male	24 (52.2%)
Smoking status	
Never	5 (10.9%)
Former	25 (54.3%)
Current	14 (30.4%)
Ever	2 (4.3%)
Current status	
Alive	15 (32.6%)
Dead	31 (67.4%)
Recur	
No	30 (65.2%)
Yes	16 (34.8%)
Stage	
IIB	1 (2.2%)
IIIA	35 (76.1%)
IIIB	8 (17.4%)
IV	2 (4.3%)
Grade	
Well	8 (17.4%)
Moderate	28 (60.9%)
Poor	10 (21.7%)
Surgery	
No	2 (4.3%)
Yes	44 (95.7%)
Chemotherapy	
No	19 (41.3%)
Yes	27 (58.7%)
Radiation therapy	
No	27 (58.7%)
Yes	19 (41.3%)
Treatment groups	
Chemotherapy	14 (30.4%)
Radiation	6 (13.0%)
Chemoradiation	13 (28.3%)
None	13 (28.3%)

### Total RNA isolation from tumor tissues

Total RNA was isolated from the flash frozen lung tumor tissues using Allprep DNA/RNA Mini Kit (Qiagen), as per manufacturer’s protocol. Briefly, five 10 µm sections of tumor tissue were homogenized in RLT plus buffer supplemented with 1% β-mercaptoethanol. The lysed mixture was centrifuged at 13000 rpm for 10 minutes to precipitate any remaining tissue debris. The clear supernatant was carefully collected, transferred to the AllPrep DNA spin column, and centrifuged for 30 s at 13,000 rpm. The flow-through was collected for RNA isolation, and the AllPrep DNA spin column was used for DNA isolation to be used elsewhere. For RNA isolation, flow-through was mixed with an equal volume of 70% ethanol, and the mixture was filtered through an RNAeasy column by centrifugation at 10000 rpm for 30 seconds. The flow-through was discarded, and the column was further washed using two changes of 700 µL RW wash buffer, and once with 500 µL RPE wash buffer. Finally, the column-bound RNA was eluted using 30 µL RNAse free water. The eluted RNA was stored at -80°C until further use.

### Quantitative Polymerase Chain Reaction

Total RNA obtained from the above step was transcribed into complementary DNA (cDNA) using SuperScript™ III First-Strand Synthesis System (Invitrogen) following the manufacturer’s protocol. Briefly, 1 µg of total RNA from each sample was mixed with 50 µM oligo[dT]_20_ primer and 10 mM dNTP mix. The mixture was incubated at 65°C for 5 min followed by 1 min at 4°C. Next, 10 µl of cDNA synthesis mix was added to each RNA/primer mixture and was incubated at 50°C for 50 min, and the reaction was terminated by heating at 85°C for 5 min followed by cooling the sample at 4°C. Finally, 1 µL of the cDNA was amplified using probe-specific primers (**Table 2**), and Power SYBR Green mastermix in an ABI 7500 real-time PCR system using the following protocol: 1x 10 min at 95°C, 40x (30 sec at 95°C, 1 min at 60°C) and hold at 4°C. ΔC_t_ values were calculated by subtracting C_t_ values for β-actin from each sample’s respective genes.

**Table 2:** Primer sequences used in this study.

PD-L1	Forward	GGCATCCAAGATACAAACTCAA
	Reverse	CAGAAGTTCCAATGCTGGATTA
DRD2	Forward	AGACCATGAGCCGTAGGAAG
	Reverse	GCAGCCAGCAGATGATGA
CHRNA5	Forward	CTGCTAGGCTGAGGCTGCT
	Reverse	ACAAAACGAGGGCAGACG
CHRNA7	Forward	CCAATGACTCGCAACCACT
	Reverse	TGTTGGTGGTTAAAACTTGGTTC
CHRNA9	Forward	GGCCATGACTGTATTTCAGCTA
	Reverse	GGCCATCGTGGCTATGTAGT
ACTB	Forward	CATGTACGTTGCTATCCAGGC
	Reverse	CTCCTTAATGTCACGCACGAT

### Statistical analysis

We used R (version 3.6.0) and Graphpad Prism (version 9) for data analyses and presentation. We did not perform sample size calculation for this retrospective study due to its descriptive nature. Categorical data were summarized as frequency counts and percentages, and were compared using Fisher’s exact test. Continuous data were summarized as mean, standard deviation, or median and interquartile ranges (IQR), compared using the Wilcoxson rank-sum test. Time‐to‐event data were summarized using the Kaplan‐Meier method and compared using log‐rank tests. We used Cox proportional hazards model for multivariable analyses of potential prognostic indicators with p<0.05 in univariate analysis. All Cox proportional hazards regression results are presented as hazard ratios (HRs), 95% confidence intervals (95% CI) for the HR, and corresponding p values. The proportionality assumption was assessed graphically using log (−log) plots and quantitatively using the Z statistic. All statistical tests were two-sided. P<0.05 was considered statistically significant.

## Results

### Patient characteristics

Forty-six NSCLC patients were included in the study and their demographic and clinical characteristics are summarized in **Table 1**. The median age of the patients was 65. Patients were grouped according to their gender, smoking status, vital status, recurrence, tumor stage, histologic grade, and treatment post-surgery. Ever smokers were those who were not clearly identified as current or former smokers at the time of lung cancer diagnosis.

### PD-L1, DRD2, and nAChR expression and selected patient characteristics

**Figure 1:**
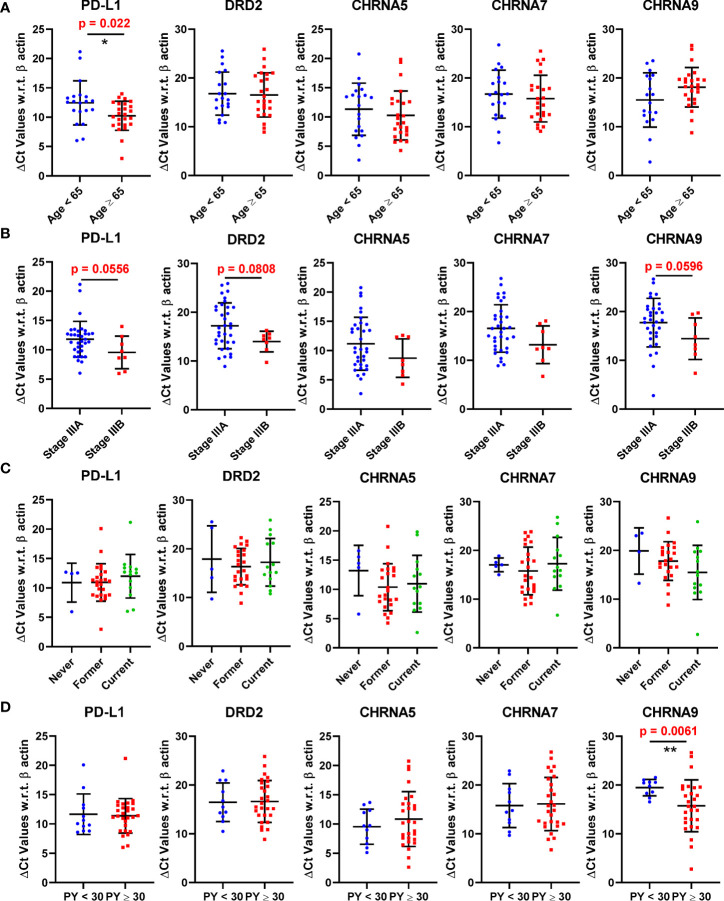
Expression of PD-L1, DRD2, CHRNA5, CHRNA7, and CHRNA9 mRNAs represented as ΔCt values in patients stratified based on age **(A)**, Stage **(B)**, Smoking status **(C)**, and pack years **(D)**. A lower ΔCt value means the gene expression is higher. * and ** denote p<0.05 and p<0.01 respectively.

The expression of PD-L1, DRD2 and three nAChR genes encoding α5, α7, and α9 nAChR respectively (CHRNA5, CHRNA7, and CHRNA9) were analyzed against selected patient characteristics such as age, stage, smoking status, and pack-years (**Figures 1A–D**). For the graphical presentation, the patients were divided based on their median age of diagnosis, which was 65 years. Interestingly patients with an age of diagnosis greater than or equal to 65 showed a statistically significant (p = 0.022) lower mean ΔCt value (and hence a higher mean expression) for PD-L1 than patients with an age of diagnosis less than 65 (**Figure 1A**). DRD2, CHRNA5, CHRNA7, or CHRNA9 did not show any significant difference between these patient cohorts. Stage IIIB patients showed lower mean ΔCt values or higher mean expressions than stage IIIA patients for each of the genes; however only PD-L1 (p = 0.0556) and CHRNA9 (p=0.0596) were close to reaching statistical significance (**Figure 1B**). Similarly, we did not observe a significant difference in expressions among never, former, and current smokers (**Figure 1C**). However, patients with pack-years greater than or equal to 30 showed a highly significant lower mean ΔCt value or higher mean expression of CHRNA9 than patients with PY less than 30 (p = 0.0061) (**Figure 1D**).

### Correlation among PD-L1, DRD2, and nAChR expression

**Figure 2:**
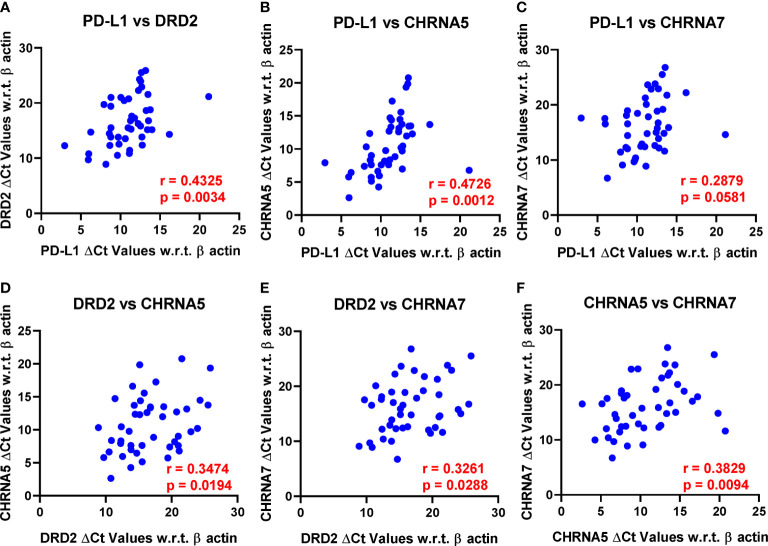
Pearson’s correlation analysis was performed to analyze the correlation between PD-L1, DRD2, CHRNA5, CHRNA7, and CHRNA9 mRNA expression represented as ΔCt values. Only those reaching or close to reaching statistical significance were included. r, Pearson’s correlation coefficient. **(A)** PD-L1 vs DRD2, **(B)** PD-L1 vs CHRNA5, **(C)** PD-L1 vs CHRNA7, **(D)** DRD2 vs CHRNA5, **(E)** DRD2 vs CHRNA7, and **(F)** CHRNA5 vs CHRNA7.

PD-L1 expression showed strong positive correlation with DRD2 (p = 0.0034) and CHRNA5 (p = 0.0012) and was close to reaching statistical significance with CHRNA7 (p = 0.0581) (**Figures 2A–C**). In addition to PD-L1, DRD2 also showed positive correlation with CHRNA5 (p = 0.0194) and CHRNA7 (p = 0.0288) (**Figures 2D, E**). Furthermore, CHRNA5 and CHRNA7 showed strong positive correlation (p = 0.0094) (**Figure 2F**). We also checked the association between the above genes in The Cancer Genome Atlas Lung Adenocarcinoma (TCGA-LUAD) database using the TIMER portal (https://cistrome.shinyapps.io/timer/). Interestingly, there we found statistically significant correlation between CHRNA5/CHRNA9 (p = 1.02e-06), CHRNA5/PD-L1 (p = 3.64e-03), CHRNA7/PD-L1 (p = 3.72e-05), CHRNA7/DRD2 (p = 2.09e-05), and CHRNA9/DRD2 (p = 4.11e-05) (**Supplementary Figure 1**). However, it is to be noted that expression of these genes may be affected by the stage, population, or treatment which may significantly vary between our study and TCGA-LUAD database. But more importantly, the notion that the expression of the nAChRs, DRD2, and PD-L1 might have some level of correlation was certainly substantiated from these data.

### Prognostic values of PD-L1, DRD2, and nAChR expression

We further evaluated the association of PD-L1, DRD2, and nAChR expression with Overall survival (OS) and Relapse-free survival (RFS). OS was defined as the time interval between the date of diagnosis and the date of death or last follow-up (censored). RFS was defined as the time interval between the date of surgical resection and the date of recurrence, or the date of death or last follow-up if no recurrence occurred (censored). We excluded three extreme outliers (one in CHRNA5 and two in CHRNA9) from the analyses. We excluded patients with stage IIB and IV disease (n=3) from the survival analysis. Due to the small sample size, we regrouped treatment categories for survival analysis to avoid overfitting. Ever smokers were combined with current and former smokers for survival analysis. Here, we treated age and expressions of the genes as continuous variables instead of dichotomized for the analyses since it provides a higher statistical power. As shown in **Table 3**, older age was associated with poor OS (HR 1.05, 95% CI 1.00-1.09, *p*=0.038); lower DRD2 expression (HR 0.91, 95% CI 0.84-1.00, *p*=0.040) and lower CHRNA7 expression (HR 0.92, 95% CI 0.85-0.99, *p*=0.037) were associated with better OS. Lower CHRNA9 expression seemed to be associated with better OS, but did not reach statistical significance (HR 0.95, 95% CI 0.89-1.01, *p*=0.093). Based on model selection criteria and the significant correlation between DRD2 and CHRNA7, we include age and CHRNA7 for multivariable survival analysis. Older age was independently associated with poor OS (HR 1.05, 95% CI 1.01-1.10, *p*=0.014); lower CHRNA7 expression was independently associated with better OS (HR 0.90, 95% CI 0.82-0.98, *p*=0.017). Lower CHRNA7 expression seemed to be associated with better RFS, but did not reach statistical significance (HR 0.94, 95% CI 0.87-1.01, *p*=0.08).

**Table 3:** Prognostic values of biomarkers, clinical factors, histopathological factors, and treatment in overall survival (OS) and relapse-free survival (RFS).

Variables		HR (univariate for OS)	HR (multivariable for OS)	HR (univariate for RFS)
Age at diagnosis		1.05 (1.00-1.09), p=0.038	1.05 (1.01-1.10), p=0.014	1.03 (0.99-1.07), p=0.116
PD-L1 expression		1.03 (0.91-1.17), p=0.612	–	1.05 (0.94-1.17), p=0.383
DRD2 expression		0.91 (0.84-1.00), p=0.040	–	0.95 (0.88-1.03), p=0.202
CHRNA5 expression		0.93 (0.86-1.01), p=0.096	–	0.96 (0.90-1.03), p=0.286
CHRNA7 expression		0.92 (0.85-0.99), p=0.037	0.90 (0.82-0.98), p=0.017	0.94 (0.87-1.01), p=0.080
CHRNA9 expression		0.95 (0.89-1.01), p=0.093	–	0.97 (0.91-1.03), p=0.280
Gender	female	–	–	–
	male	0.85 (0.42-1.76), p=0.670	–	1.17 (0.59-2.31), p=0.650
Smoking status	never	–	–	–
	former	3.12 (0.72-13.64), p=0.130	–	1.47 (0.49-4.39), p=0.494
	current	3.24 (0.70-14.92), p=0.131	–	1.44 (0.45-4.55), p=0.539
	ever	7.50 (1.02-55.05), p=0.048	–	6.63 (1.12-39.21), p=0.037
Smoking status	never	–	–	–
	ever	3.28 (0.78-13.86), p=0.106	–	1.51 (0.53-4.32), p=0.443
Stage	IIIA	–	–	–
	IIIB	1.60 (0.71-3.60), p=0.259	–	1.34 (0.60-2.96), p=0.472
Grade	well differentiated	–	–	–
	moderately differentiated	2.05 (0.69-6.07), p=0.195	–	1.65 (0.62-4.43), p=0.317
	poorly differentiated	1.97 (0.57-6.77), p=0.282	–	1.32 (0.43-4.04), p=0.632
Chemotherapy	no	–	–	–
	yes	1.09 (0.52-2.30), p=0.816	–	1.57 (0.77-3.19), p=0.214
Radiation therapy	no	–	–	–
	yes	0.90 (0.43-1.86), p=0.776	–	1.18 (0.60-2.33), p=0.625
Treatment group	none	–	–	–
	chemotherapy	1.22 (0.45-3.28), p=0.698	–	1.44 (0.56-3.67), p=0.445
	radiation therapy	1.00 (0.29-3.47), p=0.995	–	0.98 (0.29-3.29), p=0.971
	chemotherapy and radiation therapy	1.00 (0.38-2.64), p=1.000	–	1.67 (0.68-4.09), p=0.264
Treatment group	none	–	–	–
	Radiation therapy	1.00 (0.29-3.48), p=0.999	–	0.98 (0.29-3.29), p=0.969
	Chemotherapy with or without radiation therapy	1.09 (0.46-2.60), p=0.842	–	1.56 (0.69-3.51), p=0.286
Treatment group	none + radiation therapy	–	–	–
	Chemotherapy with or without radiation therapy	1.09 (0.52-2.30), p=0.816	–	1.57 (0.77-3.19), p=0.214

## Discussion

The implications of different nAChR expression and their polymorphisms in lung cancer cell proliferation, apoptosis, angiogenesis, and invasion have been previously reported by several groups (18, 71–76). Among them, homomeric α7 nAChR (composed of five identical α7 subunits expressed from the CHRNA7 gene) is the most widely implicated in nicotine-mediated proliferation, angiogenesis, and metastasis in NSCLC (77–81). Additionally, both α5 and α9 nAChRs (encoded by CHRNA5 and CHRNA9 genes, respectively) have been associated with NSCLC (77, 82–85). A recent study using lung adenocarcinoma (ADC) patient samples revealed that α5-nAChR expression is correlated with the clinicopathological parameters such as T and N stages but not with age or sex (82). Another study with lung squamous cell carcinoma (SQCC) and ADC showed that α5-nAChR expression is significantly higher in tumors than in adjacent normal lung tissue (86). Interestingly α7-nAChR was significantly higher in SQCC than normal tissue but not in ADC in this study. However, another study had shown that α7-nAChR expression was significantly higher in both SQCC and ADC (87). Both α5- and α7-nAChR were significantly associated with unfavorable prognosis in ADC, but only α7-nAChR showed a significant correlation with prognosis in SQCC. Unfortunately, several important factors, including treatment modalities such as chemotherapy and radiation therapy, were missing in these studies, making the conclusion challenging to interpret.

The correlation of nAChRs and PD-L1 has been shown by *in vitro* studies in several human cell lines, including bronchial epithelial cells, HepG2 cells, melanoma and breast cancer cells (88–91). Furthermore, an *in vitro* study showed that chronic nicotine exposure could increase α1-nAChR and PD-L1 expressions in a lung adenocarcinoma cell line (92). Another recent study showed that coexpression of α5-nAChR and PD-L1 are associated with a worse prognosis in patients with lung adenocarcinoma (93). However, the correlation between PD-L1 and DRD2 has not been well-investigated in context to human cancer except for one recent study where Paliperidone (a DRD2 antagonist) reduced PD-L1 expression in glioblastoma cells and increased survival in a mouse model of glioblastoma (94).

Nicotine typically induces the release of dopamine in the brain *via* activation of the nAChR receptors in the central nervous system, but the released dopamine cannot cross blood-brain barrier (71). Interestingly, dopamine can also be synthesized in the peripheral nerves and released into circulation in response to stress, exercise, or hypovolemia (95). Nonetheless, a plausible role of nicotine or nAChRs in peripheral dopamine synthesis may not be ruled out, especially since correlative analyses from our data as well as TCGA database showed positive correlation between some of the nAChRs and DRD2 in lung adenocarcinoma patients (**Figure 2** and **Supplementary Figure 1**).

Nicotinic acetylcholine receptors such as a5, a7, or a9 have been shown to express in various immune cells where they regulate the secretion of immunomodulatory cytokines and immune response (96). Interestingly, nicotine-mediated activation of both a5 and a9 nAChRs have been shown to upregulate PD-L1 expression in cancer cells *via* STAT3 signaling pathways (90, 93), so a positive correlation of nAChRs with PD-L1 may be expected (**Supplementary Figure 2**). Surprisingly, the nAChRs correlated with DRD2 or PD-L1 are different between our study and TCGA database, but the difference may be attributed to the difference in stages, patient population, or tumor-infiltrating immune cells. For instance, differential tumor infiltration of immune cells having varied expression of nAChRs or DRD2 can affect their correlation in the tumor samples.

DRD2 is also expressed on the surface of a variety of immune cells and has been implicated in the regulation of immune cell activity (97). DRD2 antagonism has been reported to induce immune cell proliferation and activation in preclinical studies, whereas DRD2 activation suppressed the function of natural killer cells. DRD2 stimulation has also been shown to inhibit proliferation and cytokine production in activated T cells. DRD2 antagonists could also induce M1-polarization of macrophages and decrease PD-L1 expression in cancer cells *via* inhibition of ERK and STAT3 signaling pathways (94, 98). Consequently, a positive correlation between DRD2 and PD-L1 may not be improbable after all (**Supplementary Figure 2**).

Our study demonstrated positive correlations among PD-L1, DRD2, and nAChR in tumor samples from 46 NSCLC patients at our institution. Our study indicates a possible underlying mechanism that DRD2 and nAChR involved pathways may affect the tumor immune microenvironment, leading to the expression of PD-L1. It remains unknown whether DRD2 and nAChR share the same signaling pathways or play a synergetic role in tumorigenesis and therefore requires further investigation. In addition, given the increased roles of anti-PD-1/anti-PD-L1 immunotherapies in early-stage and advanced-stage NSCLC, our data imply that DRD2 and nAChR might be potential molecular biomarkers along with PD-L1 to guide treatment decisions in NSCLC patients in the future. Furthermore, targeting nAChR or DRD2 may be a potential therapeutic strategy to alter PD-1/PD-L1 pathway that can benefit those NSCLC patients who are refractory to immunotherapy.

We found that lower expressions of DRD2 and CHRNA7 are associated with a slightly better OS. Previous studies showed that a high expression of CHRNA7 is associated with an unfavorable prognosis in NSCLC (45). Of note, most patients studied in this cohort are stage I and II. We observed a similar result in our cohort of stage III NSCLC patients, in which lower CHRNA7 expression is independently associated with better OS, although the difference is small (HR = 0.90). Whether similar trend can be observed in stage IV NSCLC patients and this is associated with any clinical significance needs to be further evaluated. We did not observe statistically significant differences in RFS regarding the expression of each gene tested. This may be due to the small size of our cohort. Whether DRD2 or nAChR affects or has predictive value of the response to systemic therapy, such as chemotherapy or immunotherapy, remains unknown and warrants further investigation.

We did not observe a significant difference in PD-L1, DRD2, and nAChR expressions among never, former, and current smokers. Nevertheless, patients with more pack-year (>30) smoking history have significantly higher CHRNA9 expression, but not CHRNA5 or CHRNA7 expression. This indicates a possible unique smoking-related upregulating mechanism on CHRNA9 gene expression, which requires further elucidation.

Although our study suggests a probable connection of DRD2 with nAChR and PD-L1 in lung cancer for the first time, there are several limitations in our study. First, we only examined gene expression at the mRNA level by using qPCR due to the limited availability of tumor tissues. Whether the observed trends hold true at the protein level needs further validation. Additionally, immunohistochemistry data would have been potentially useful in determining the spatial expression of these markers in the tumor microenvironment. This is important since it would delineate the effect of different tumor-infiltrating immune cell populations with varied nAChR, DRD2 or PD-L1 expression on the prognosis. Second, our cohort has a relatively small size. This limits the statistical power to detect the difference in targeted gene expression between subgroups, such as smoking status. Furthermore, our study only includes localized NSCLC patients who received surgery. It is important to extend our analysis to a large-scale study that includes patients who are not surgical candidates or at stage IV to evaluate DRD2 and nAChR’s role in systemic therapy.

## Conclusion

In conclusion, we investigated the expression pattern and prognostic value of PD-L1, DRD2, and three nAChR family members in NSCLC using surgical samples obtained from 46 patients. We found strong positive correlations between PD-L1/DRD2, PD-L1/CHRNA5, and CHRNA5/CHRNA7 and a weak positive correlation between DRD2/CHRNA5 and DRD2/CHRNA7 at the mRNA level. CHRNA7 was an independent prognostic factor in surgically resected stage III NSCLC patients. Further studies using large-scale cohorts including patients at different stages and receiving various treatments are warranted.

## Data availability statement

The original contributions presented in the study are included in the article/**Supplementary Material**. Further inquiries can be directed to the corresponding author.

## Ethics statement

The studies involving human participants were reviewed and approved by The Mayo Clinic Institutional Review Board. The patients/participants provided their written informed consent to participate in this study.

## Author contributions

DM and SC conceived the idea and supervised overall research. PY and AM arranged the patient sample collection. KP designed the experiments, analyzed, and interpreted the data. TH performed the experiments. HX and PY performed statistical analyses. KP, TH, HX, and SL wrote the original manuscript. DM, SC, PY and YL revised the article. All authors contributed to the article and approved the submitted version.

## Funding

This work was supported by NIH grants CA150190, CA78383, Florida Department of Health Grant #20K02, and Cancer Research Chair Fund #3J (DM), the DBT/Wellcome Trust India Alliance Fellowship [grant number IA/S/18/2/504021] awarded to SC, and CSIR-Mayo Clinic Collaboration for Translation Research Program for support to TH.

## Acknowledgments

The authors thank Dr. Jin Jen for providing critical resources.

## Conflict of interest

The authors declare that the research was conducted in the absence of any commercial or financial relationships that could be construed as a potential conflict of interest.

## Publisher’s note

All claims expressed in this article are solely those of the authors and do not necessarily represent those of their affiliated organizations, or those of the publisher, the editors and the reviewers. Any product that may be evaluated in this article, or claim that may be made by its manufacturer, is not guaranteed or endorsed by the publisher.

## Supplementary material

Supplementary Figure 1 Pearson’s correlation analysis was performed to analyze the correlation between PD-L1 (CD274), DRD2, CHRNA5, CHRNA7, and CHRNA9 mRNA expression from TCGA-LUAD database.

Supplementary Figure 2 A schematic diagram depicting plausible connection between nAChR, DRD2, and PD-L1. Both nAChR and DRD2 activation by their respective ligands induce PD-L1 expression *via* STAT3 signaling mechanism. However, it is not clear how nAChRs are involved in peripheral DRD2 signaling, although they are known to be involved in dopamine synthesis in the brain. Created with BioRender.com.
