# Supplementary figures and images for: Expression, correlation, and prognostic significance of different nicotinic acetylcholine receptors, programed death ligand 1, and dopamine receptor D2 in lung adenocarcinoma
Source: Front Oncol. 2022 Aug 22;12:959500. doi: 10.3389/fonc.2022.959500 (PMC9441878; doi:10.3389/fonc.2022.959500)

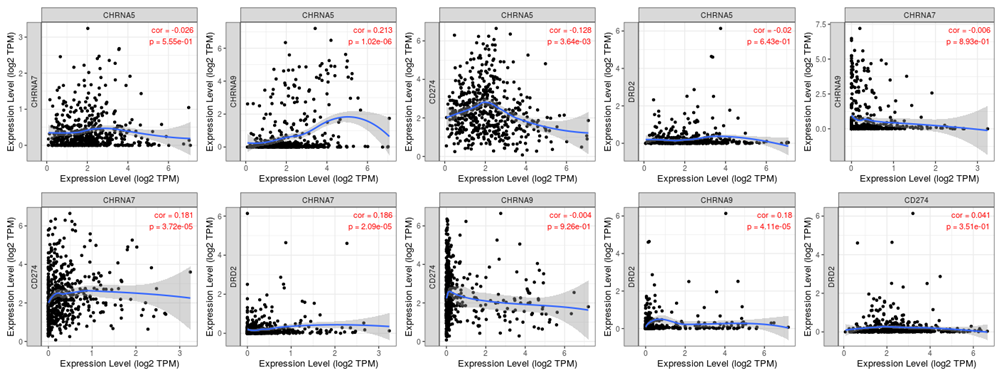

Supplement: Supplementary Figure 1 — Pearson’s correlation analysis was performed to analyze the correlation between PD-L1 (CD274), DRD2, CHRNA5, CHRNA7, and CHRNA9 mRNA expression from TCGA-LUAD database. [file Image_1.tif]

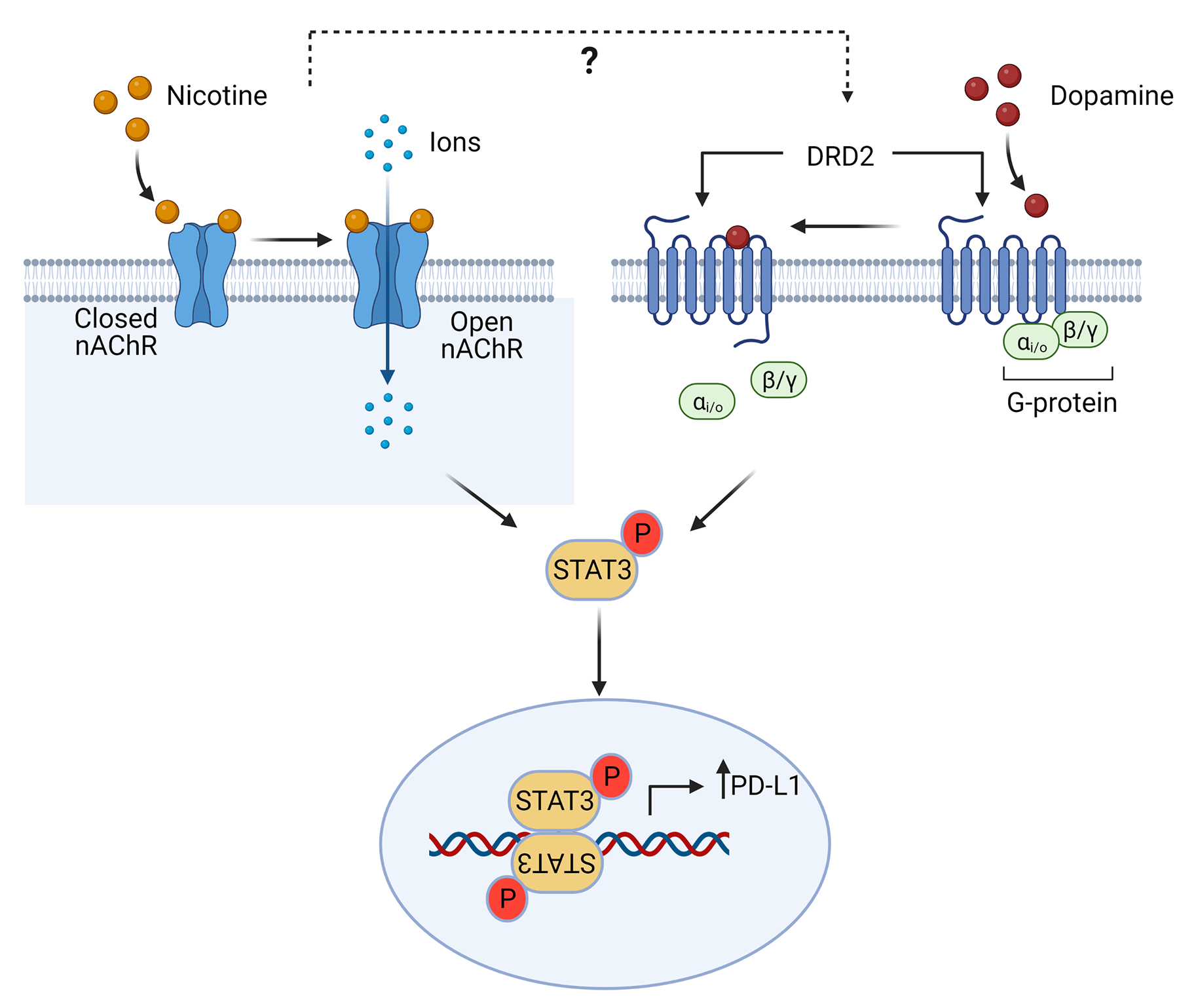

Supplement: Supplementary Figure 2 — A schematic diagram depicting plausible connection between nAChR, DRD2, and PD-L1. Both nAChR and DRD2 activation by their respective ligands induce PD-L1 expression via STAT3 signaling mechanism. However, it is not clear how nAChRs are involved in peripheral DRD2 signaling, although they are known to be involved in dopamine synthesis in the brain. Created with BioRender.com. [file Image_2.tif]
